# Few-Layer Graphene-Based Optical Nanobiosensors for the Early-Stage Detection of Ovarian Cancer Using Liquid Biopsy and an Active Learning Strategy

**DOI:** 10.3390/cells14050375

**Published:** 2025-03-04

**Authors:** Obdulia Covarrubias-Zambrano, Deepesh Agarwal, Joan Lewis-Wambi, Raul Neri, Andrea Jewell, Balasubramaniam Natarajan, Stefan H. Bossmann

**Affiliations:** 1 Department of Cancer Biology, University of Kansas Medical Center, Kansas City, KS 66160, USA; ocovarrubias@kumc.edu (O.C.-Z.); jlewis-wambi@kumc.edu (J.L.-W.); rnerisierra@kumc.edu (R.N.); 2 Department of Electrical and Computer Engineering, Kansas State University, Manhattan, KS 66506, USA; deepesh@ksu.edu (D.A.); bala@ksu.edu (B.N.); 3 Department of Obstetrics and Gynecology, University of Kansas Medical Center, Kansas City, KS 66160, USA; ajewell@kumc.edu

**Keywords:** graphene-based nanobiosensors, biomarkers, liquid biopsy, ovarian cancer detection, protease activity, biophotonics, hierarchical decision structure

## Abstract

Ovarian cancer survival depends strongly on the time of diagnosis. Detection at stage 1 must be the goal of liquid biopsies for ovarian cancer detection. We report the development and validation of graphene-based optical nanobiosensors (G-NBSs) that quantify the activities of a panel of proteases, which were selected to provide a crowd response that is specific for ovarian cancer. These G-NBSs consist of few-layer explosion graphene featuring a hydrophilic coating, which is linked to fluorescently labeled highly selective consensus sequences for the proteases of interest, as well as a fluorescent dye. The panel of G-NBSs showed statistically significant differences in protease activities when comparing localized (early-stage) ovarian cancer with both metastatic (late-stage) and healthy control groups. A hierarchical framework integrated with active learning (AL) as a prediction and analysis tool for early-stage detection of ovarian cancer was implemented, which obtained an overall accuracy score of 94.5%, with both a sensitivity and specificity of 0.94.

## 1. Introduction

### 1.1. Ovarian Cancer Is a Silent Killer

Ovarian cancer (OC), one of the cancer types known as a “silent killer”, is the most lethal of all gynecologic malignancies and the fifth deadliest cancer type in the US [1]. Globally, OC is the seventh most diagnosed and the eighth leading cause of cancer-related mortality among women [2,3,4,5]. According to estimates by the Surveillance, Epidemiology, and End Results Program of the NCI, 20,890 new OC cases and 12,740 estimated deaths will occur in 2025 [6]. OC is a deadly disease with no effective screening. Approximately 70% of patients with OC are diagnosed at an advanced stage, with associated poor prognosis, even after aggressive and immediate treatments [7]. The high mortality rate of OC can be attributed to the difficulty in detecting the disease, particularly at the early stages when clinical interventions are most effective, hence the term “silent killer” [8]. Routine screening for early detection has shown considerable benefits for many forms of cancer. For example, since the introduction of the Papanicolaou (Pap) test, the incidence and mortality of cervical cancer in US screened populations have declined by over 75% [9]. Similarly, colonoscopy screening was shown to be associated with a 70% mortality risk reduction for colorectal cancer [10]. Unfortunately, in the case of ovarian cancer, there is no universal screening program for early detection [11]. Currently, there are two ways to screen for ovarian cancer before it causes symptoms or shows up during a routine gynecologic exam. One is a blood test for elevated levels of a protein called cancer antigen 125 (CA-125) [12], and the other is an ultrasound of the ovaries. Unfortunately, neither technique has been shown to save lives when used in women of average risk. Consequently, routine screening for ovarian cancer is, at present, not recommended by the United States Preventative Services Task Force (USPSTF) since “the potential harms outweigh the potential benefits” [11,12,13,14]. Hence, there is an urgent need for accurate screening and diagnostic tests for OC at an early stage using optical graphene-based nanobiosensor biomarkers.

The Bossmann/Covarrubias group has developed multiple nanobiosensors for protease and arginase activity measurements [15,16,17,18,19]. The detection of early cancers (breast [16], pancreatic [17], and lung [20]) was achieved by monitoring crowd responses from panels of proteases that were selected from the NIH Gene Expression Omnibus (GEO) [21]. Different enzymatic activity levels in serum from cancer patients and age-matched healthy individuals have been successfully monitored to differentiate between cancer-free patients, early-state cancer patients, and patients with distant cancers.

### 1.2. Explosion Graphene Nanobiosensors

Graphene (G) was first isolated and discovered in 2004. It has a unique combination of extraordinary properties, such as electrical and thermal conductivity, mechanical strength, and optical properties [22]. It is important to consider that for the development of optical nanobiosensors (NBSs), the (photo)physical and chemical properties of graphenes from various sources depend on their production methods [23]. Most commercial graphene is produced by the exfoliation of graphite under oxidative conditions [24] and subsequent chemical reduction [25] or by means of chemical vapor deposition (CVD). Newer methods comprise plasma-based G synthesis [26] and flash graphene [27]. What all of these methods have in common is that they produce microscopic or larger graphene flakes varying in the size and number of stacked surfaces [23]. This nanobiosensor technology requires well-defined graphene that is composed of stacked nanoscopic graphene nanosheets, virtually without defects (with a very high content of sp^2^-hybridized carbons), and intrinsically fractal. The only available source to date is explosion graphene, which was originally discovered by Dr. Sorensen [28] at Kansas State University and is now produced by HydroGraph Clean Power in Manhattan, KS [29]. Explosion graphene is synthesized via gas-phase explosion reactions [28]. Because of its few-layered structure (n = 5–8), explosion graphene and its surface-modified derivatives quench all luminescence occurring from the graphene itself and from tethered fluorophores within a 20nm radius [30]. Together with its large BET (Brunauer–Emmett–Teller) surface (150 nm^2^/g) [28,31], explosion graphene is the ideal material for the design of fluorescence-based NBSs, which are 100 times more sensitive than competing technologies, such as immunoprecipitation and graphene oxide-based optical sensors [32,33].

**Figure 1:**
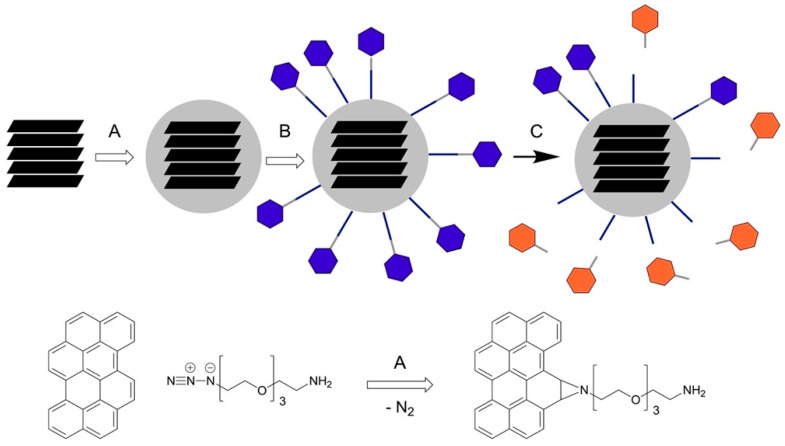
(**A**) Coating of graphene with TEG4amine. (**B**) Attachment of consensus sequence and attached fluorophore (TCPP). (**C**) Enzymatic activation of a fluorescence readout.

In 2020, Hawkeye Bio LLC [34], a development-stage medical technology company based in Torrance, California, gained interest in this technology for early lung cancer detection. In close collaboration with Hawkeye Bio, we transitioned our nanobiosensor technology from using Fe/Fe_3_O_4_ core/shell nanoparticles and a FRET donor/acceptor pair [15,16,17,18,19] to using few-layer graphene nanosheets as the sole quencher in conjunction with a consensus sequence and a fluorophore [35]. Explosion graphene nanobiosensor (G-based NBS) technology has the capacity to reach the sub-femtomolar (10^−15^ mol/L) limit of detection levels [35]. Compared with Fe/Fe_3_O_4_ nanoparticles, few-layer graphene nanobiosensors (G-NBSs) are highly water/buffer dispersible after chemical modification. They show superior long-term stability. Figure 1 shows the synthesis and activation of G-NBSs (A), and the key chemical transformation step of the graphene surface is shown in (B).

Liquid biopsies have become a powerful and promising group of methods that could potentially replace solid tumor tissue biopsies, which are the gold standard for tumor profiling to date [36].

Tissue biopsies are invasive, risky, and, many times, not easily obtained based on the tumor/tissue location within the body [37]; these are just a few limitations for what are considered the best tumor profiling methods currently available. Liquid biopsies consist of the detection of cancer-derived components in the bodily fluids of patients (blood, urine, serum, saliva, etc.) to potentially provide information about possible tumors present and metastases [36]. This non-invasive detection method can be utilized not only for a first cancer diagnosis but also for follow-up check-ups of cancer survivors to monitor for cancer recurrence, which is the case for many cancer survivors. For these reasons, liquid biopsies have a great potential for clinical care in oncology [36].

Cancer biomarkers [38] are biological molecules (enzymes, antibodies, peptides, etc.) that are present in bodily fluids or tissue and can be used for a cancer diagnosis based on their expression and activity. This study focuses on proteases [39], enzymes that degrade proteins. Proteases regulate signaling pathways and play essential roles in cancer progression and metastasis [40,41]. Furthermore, the proteolytic network interacts with other important signaling pathways in tumor biology, involving chemokines, cytokines, and kinases [39]. Over 600 human proteases are responsible for regulating processes, which, many times, are dysregulated in tumors [42]. For example, matrix metalloproteinases (MMPs) are a family of proteases that can degrade the extracellular matrix (ECM), connective tissues, and basement membranes and are over-expressed in both precancerous and cancerous cells; they promote tumor progression and play important roles in cancer invasion and metastasis [43,44]. Just like MMPs, cathepsins (CTSs) and a disintegrin and metalloproteinase (ADAM) are either over- or under-expressed in various cancer types, playing essential roles in cancer growth, progression, and metastasis [41,45,46]. The NCBI GEO database [47] contains high-throughput screening genomics data from microarray or RNA-Seq profiling. It enables the preselection of candidate proteases with significant over- and/or under-expression in ovarian cancer tissue. These proteases are activated by other proteases because they are players in the proteolytic network that encompasses virtually all > 600 human proteases, with the exception of caspases [39].

Active proteases can leave the tumor microenvironment by means of diffusion into the surrounding tissue, secretion by tumor cells via extracellular vesicles or direct release, and interaction with protease-specific receptors on stromal cells, which facilitate their transport from the tumor site into the bloodstream [48,49].

It has been demonstrated that the highest proteolytic activity is detected in serum samples, decreased activity is found in plasma, and the lowest activity is found in whole blood due to the presence of deactivating protein factors [50,51]. For this reason, the activity profiles of these OC-sensitive proteases were quantified in serum samples for the early detection of ovarian cancer.

It is noteworthy that there is no straightforward correlation between zymogen expression in tissue and activated proteases in serum. However, the NCBI GEO database permits an effective pre-selection of candidate proteases. In the research reported here, we found measurable protease activities for all eight genes (ADAM10/12 could not be distinguished because their active centers are too similar), which allowed us to design this study.

## 2. Materials and Methods

### 2.1. Synthesis of Peptides and Assembly of Graphene-Based Nanobiosensors

Using the NCBI gene expression omnibus [21] web tool, 4 datasets were used to identify a set of genes that were significantly different in expression levels in tissue when comparing ovarian cancer patients with a healthy control group. The top eight genes identified to have the highest differences in protease expression were selected. Each consensus sequence was synthesized following a previously established solid-phase peptide synthesis protocol [15,16,17,18,19]. Briefly, peptide synthesis started at the C-terminal end of the peptide, which was synthesized toward the N-terminal end. The synthesis started with a resin containing the first amino acid of the peptide chain, followed by a cycle of deprotection–activation coupling reactions, which was repeated for each amino acid that was added to the peptide chain. Adding one amino acid required 90 min for each cycle. Once the last amino acid was attached, TCPP (a fluorescent dye, previously synthesized as described here) [15] was then covalently linked to the peptide on the N-terminal end. Finally, the fluorescently labeled peptide was deprotected and cleaved off from the resin, dried, and stored under argon at −20 °C. The list of consensus sequences synthesized is summarized in Table 1.

A “consensus sequence” is defined as a “calculated sequence of amino acids that represents the most frequently occurring residue at each position within a set of aligned, related sequences” that occur in vivo [47]. It is noteworthy that the cross-reactivity of other proteases cannot be completely suppressed. However, principally, cross-reactivity was found to be less than 5% of the protease activity in total. If known selectivity overlapped, for instance, of MMP2 and MMP9, they were avoided in the design of the protease panel [16]. Selecting seven proteases as biomarkers for OC instead of only one protease made the panel more robust against cross-reactivity.

Consensus sequences were adapted from the MEROPS database, which is a comprehensive online resource that provides information about proteases, including their classification, structure, function, and inhibitors, essentially acting as a central repository for all things related to peptidases and their activity within biological systems [52].

### 2.2. Surface Modification of Graphene

The surface modification of graphene (G) was carried out as follows: 1.0 g of FGA-1 graphene from HydroGraph was dispersed in 50 mL of anhydrous DMF in a round-bottle flask at room temperature. The dispersion was then heated to 40 °C, followed by the addition of azide-tetraethylene glycol-amide [azideTEG4amine], which was added slowly. After allowing azideTEG4amine to adsorb on the outer surface of G for 10 min, the dispersion was heated to 80 °C and reacted for 1 h at that temperature. Then, the reaction was cooled down to room temperature. Samples were collected via centrifugation (5 min at 4500 rpm). Finally, the G-TEG4amine product was washed five times with DMF (dimethylformamide (40 mL)) and four times with anhydrous diethyl ether (40 mL) and dried and stored under argon at −20 °C. For the assembly of G-NBSs, 50 mg of G-TEG4amine was suspended in 8.0 mL of anhydrous DMF in a small vial, followed by the addition of 3.5 mg of each TCPP-labeled protease peptide along with 5 mg of EDC (1-ethyl-3-(3-dimethylaminopropyl)carbodiimide) and 5 mg of DMAP (dimethyl-amino)pyridine) [20]. The sample was sonicated for 5 min to ensure the full dispersion of everything. The assembly of G-NBS was carried out overnight at room temperature. G-NBS was collected via centrifugation (6 min at 4500 rpm), washed five times with DMF (10 mL) and four times with anhydrous diethyl ether (10 mL), and finally dried and stored under argon at −20 °C.

### 2.3. Serum Sample Information

A total of 146 serum samples were obtained from the KUMC Biospecimen Repository of the University of Kansas Cancer Center [53]. The biospecimens contained the following groups: healthy control samples (n = 50), stages I and II or localized ovarian cancer (LOC) patients (n = 46), and stages III and IV or metastatic ovarian cancer (MOC) patients (n = 50). All OC samples obtained were from patients who had just received the diagnosis and had not started any treatments yet.

### 2.4. Measurements of Protease Activity in Serum Samples

To quantify the protease activities in the serum samples mentioned above, a 0.03 mg/mL solution of G-NBS was prepared using HEPES buffer solution (25 µmol/L of HEPES buffer enriched with 10 µmol/L of Ca^2+^, Mg^2+^, and Zn^2+^ (pH 7.20)). After preparing the NBS solution, the pH was re-adjusted to 7.20 using a 0.1 M HCl solution. The following three solutions were prepared and plated in triplicate in a 96-well plate (Corning, flat-bottom polystyrene microplate, black non-binding; item#: 07201203). Solution 1: sample control (125 µL of HEPES buffer + 5 µL of collected serum). Solution 2: assay control (125 µL of protease G-NBS solution in HEPES buffer + 5 µL of HEPES buffer). Solution 3: assay (125 µL of protease G-NBS solution in HEPES buffer + 5 µL of collected serum). After all solutions were plated, each plate was incubated at 37 °C for 1 h. The fluorescence intensities were measured using a BioTek Synergy H1 plate reader (λ_ex_ = 425 ± 10 nm; λ_em_ = 650 ± 20 nm). The fluorescence intensities for both the sample control (SC) and the assay control (AC) were very low compared with the intensities for the assay, where the protease present in serum cleaved the oligopeptide tethered to time.

The activity of the graphene-based nanobiosensors used in this study was ascertained before and during the duration of this study by using pooled serum from the biorepository. Please note that the commercially available recombinant proteases display large vendor-to-vendor and batch-to-batch inconsistencies. This strategy has proven successful in a previous study [35].

### 2.5. Statistical Analysis

An F-test was performed for the raw data on each group being compared to determine if they had equal or unequal variance. Once the variance was determined, a T-test (equal variance) or Welch’s *t*-test (unequal variance) was performed to determine if significant differences were observed for each biomarker comparing cancer vs. control groups. Comparisons between the control group and cancer group included all stages. Significant differences between groups compared were reported if the *p*-value was ≤ 0.05, while a borderline significant difference was reported if the *p*-value was between 0.05 and 0.10.

### 2.6. Explainable Active Learning Framework

Here, the problem of the early-stage detection of ovarian cancer was modeled as a multi-class classification problem. The data derived from the experiments consisted of three classes, namely, “healthy”, “localized”, and “metastatic” ovarian cancer. Semi-supervised learning approaches like active learning (AL) have been demonstrated to improve label efficiency by providing flexibility to train the decision models using a limited number of labeled samples compared with traditional supervised learning approaches [54,55,56]. This is very beneficial in applications like medical diagnosis, where the labeling process may be laborious, long-standing, or expensive. This paper proposes an explainable AL decision framework for the early-stage detection of OC. AL is implemented with the following variants for the base decision model: (i) a hierarchical decision model with k-nearest neighbors (kNN) as the base classifier [57]; (ii) a 2-layer neural network with linear activations. The hierarchical decision structure breaks down the multi-class classification problem into two hierarchical steps. The first step trains a kNN model to differentiate between healthy and non-healthy samples, while the non-healthy samples are further segregated into localized vs. metastatic classes using another kNN model in the second hierarchical step. The uncertainty sampling strategy [54] is employed to select instances for annotation during the iterative querying process in the AL environment. It is worthwhile to note that the use of base decision models is just an illustration, and the proposed framework is applicable across any base classification framework. In this strategy, the instances that minimize the classifier uncertainty are selected for annotation during each query step, as indicated in Equation (1).

(1) $$x^{*}=\arg\underset{x}{\min}P_{\theta }(\hat{y}|x),\;\mathrm{where}\;\hat{y}=\arg\underset{y}{\max}P_{\theta }(y|x)$$

A proportion of 20% of the total instances in the dataset are selected randomly to create an initial labeled dataset, which is used to train an initial ML-based classification model. Furthermore, 30% of the total instances are used for querying iteratively (one query per iteration), and the classification model is updated after each query step.

To explain the decisions predicted by the proposed framework, feature relevance explanation is used as a post hoc explainability technique for the decision models. This process elucidates the internal operations of a model by calculating a relevance score for each of the associated features in the dataset. These scores measure the impact (sensitivity) a feature exerts on the model’s output. Comparing scores across multiple variables reveals the significance attributed by the model to each variable in generating its output. The feature relevance methods serve as an indirect approach to elucidate the predictions of a decision model [58]. Specifically, the SHapley Additive exPlanations (SHAP) methodology [59] is employed to address the issue of explainability. SHAP is a unified framework that assigns a significance value to each feature for a specific prediction task using a novel category of additive feature importance metrics. An additional advantage is that SHAP values remain constant unless there is a modification in the contribution of a feature. This suggests that SHAP values are model-agnostic and offer a reliable interpretation of the predictions of a decision model, regardless of alterations in its architecture or parameters. Moreover, it is robust to missing data and mitigates the risk of irrelevant features distorting the interpretation of the decision model.

## 3. Results

### 3.1. A Panel of Sensitive Proteases Permits the Detection of Stage I Ovarian Cancer

The main hypothesis of the research work here is that the quantitative activities of selected biomarkers (the panel of OC-sensitive proteases) permit the early detection of ovarian cancer. The detection of early tumors by means of protease activity measurements takes advantage of the biology of human proteases: virtually all proteases, except for caspases, form a proteolytic network and require activation by other proteases or undergo slow autocatalytic activation [42,60].

**Figure 2:**
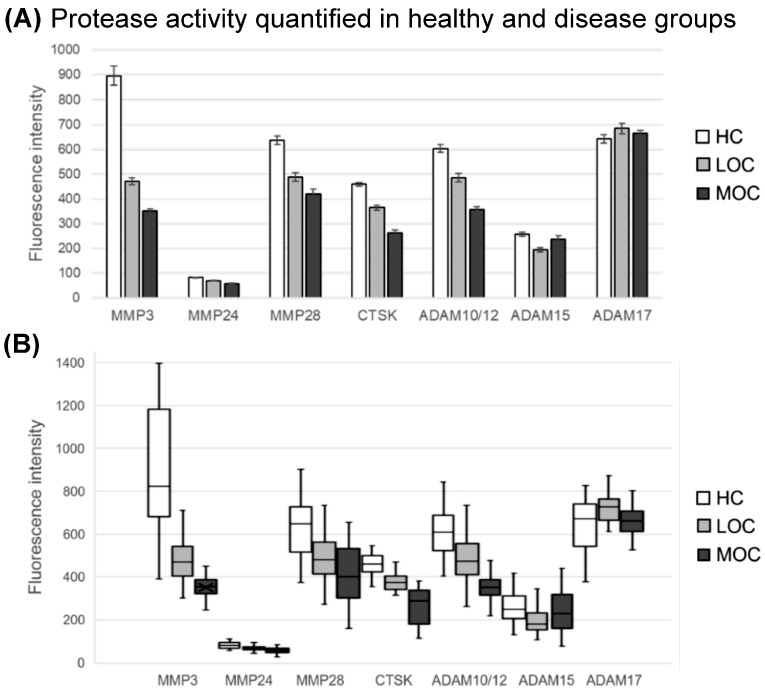
(**A**) Bar graphs and (**B**) box plots for fluorescence intensity measured for all 6 protease biomarkers in serum samples collected from ovarian cancer (LOC n = 46; MOC n = 50) and healthy control patients (n = 50). The lines shown in the box plots are the maximum and minimum values measured for each dataset analyzed. Each box has 3 lines, going from top to bottom; the 1st line is the 1st quartile, the 2nd is the median line, and the 3rd line is the 3rd quartile.

In cancer, proteases can be either mutated or misfolded, resulting in decreased proteolytic activity, or over-expressed, resulting in increased activity [32,39,41]. Activity changes in a few proteases are then amplified by the proteolytic network, leading to cancer-specific protease signatures [16,17,20]. It is our central paradigm that typical protease signatures exist for virtually all solid tumors. In this study, a panel consisting of six protease G-NBSs was used to quantify the protease activity in serum samples obtained from three different groups: localized OC, metastatic OC, and a healthy control group. Interestingly, the healthy control group resulted in the highest fluorescence intensity activity measured compared with both localized and metastatic ovarian cancer patients (Figure 2A,B), which was consistent with the GEO datafile compiled, where most of the top biomarkers were under-expressed in OC patients when compared with healthy patients. Figure 2A summarizes the mean protease intensity measured in each group for all six biomarkers, while Figure 2B shows the distribution among each group of patients tested.

The protease activity for MMP3, MMP24, MMP28, CTSK, and ADAM10/12 had the highest activity in the healthy group, followed by the LOC group, while for ADAM15, the intensity was higher for MOC vs. LOC. After running statistical analysis on these results, six out of the seven protease G-NBSs in the panel were demonstrated to have strong statistically significant differences when comparing the LOC group with both the healthy and MOC groups (values highlighted in gray), with p-values ranging from 10^−2^ to 10^−23^ (Table 2). ADAM17 was the only protease G-NBS with no significant differences for any of the OC and healthy groups analyzed. Regardless, the other six protease G-NBSs demonstrated small *p*-values. These results strengthen the hypothesis of using this panel for the early detection of ovarian cancer while differentiating early vs. late stages.

### 3.2. AL Decision Model—Performance and Explainability

**Figure 3:**
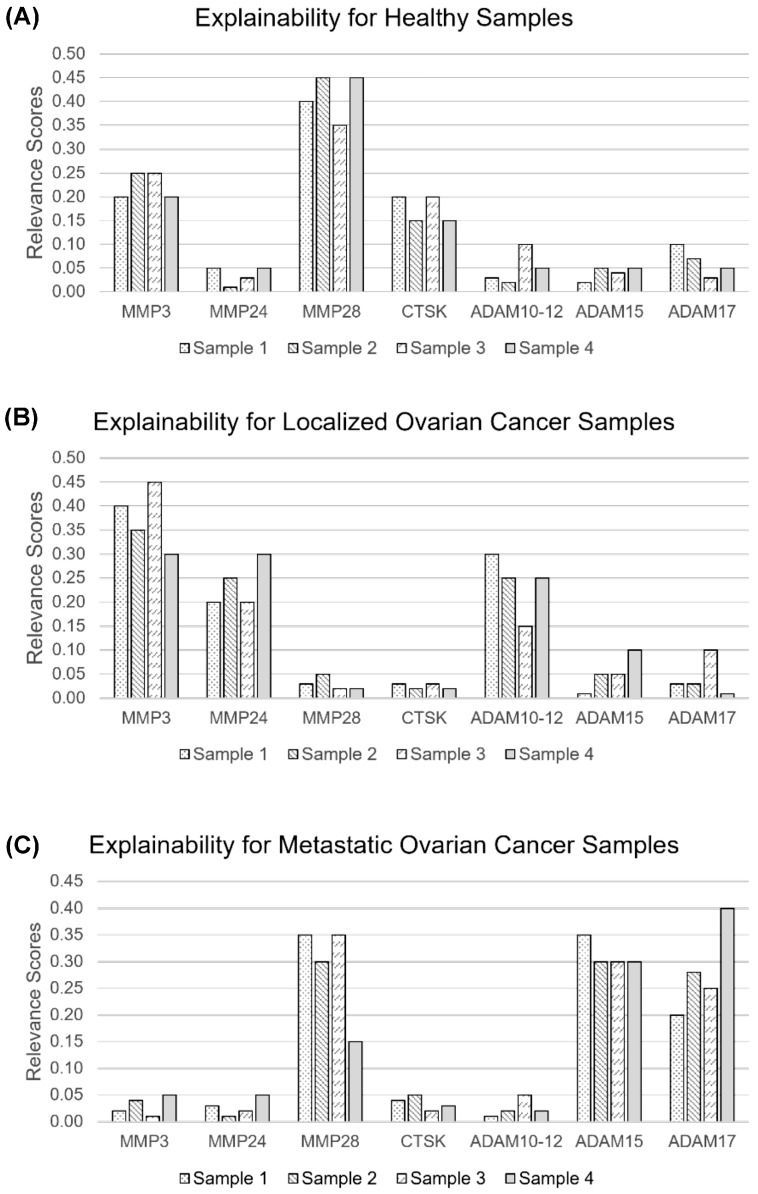
Feature relevance scores for samples in (**A**) healthy, (**B**) localized ovarian cancer, and (**C**) metastatic ovarian cancer classes.

The proposed AL methodology was evaluated by considering both variants for the base decision model: (i) the hierarchical decision framework with kNN as the base classifier and (ii) the two-layer neural network with linear activations. The corresponding confusion matrices are presented in Table 3 and Table 4, respectively. It can be observed that a classification accuracy of 93.83% was obtained when the hierarchical decision framework with kNN was employed as the base decision model within the AL environment. Meanwhile, a two-layer neural network with linear activations yielded an accuracy of 94.52%. The specificity was the same with both the variants of the decision models. Moreover, the sensitivity was slightly better with the two-layer neural network (0.94) compared with that obtained using the hierarchical decision framework with kNN (0.93) as the base decision model. This suggests that a two-layer neural network with linear activations is able to learn better representation in the proposed AL framework. The feature relevance scores were computed for all the samples in the dataset, based on the SHAP methodology elaborated in the Methods Section. A few samples from each of the classes, i.e., “healthy”, “localized”, and “metastatic” ovarian cancer are presented in Figure 3A–C, respectively.

It can be observed that the top three feature relevance scores were consistent within each class, e.g., MMP3, MMP28, and CTSK exhibited the highest feature relevance scores for samples in the “healthy” class. Similarly, MMP3, MMP24, and ADAM10-12 possessed the highest feature relevance scores for the “localized” class, and MMP28, ADAM15, and ADAM17 for the “metastatic” class. Even though ADAM17 had no significant differences between any of the OC patients or healthy control groups analyzed, for the explainability part, it was among the top three proteases to show high feature relevance scores for the metastatic class. The consistency in these feature relevance scores suggests the high importance of these features over others to explain the predictions of the decision models. This information helps the clinician judge the importance of different proteases during medical diagnosis and nudge the possibility of prescribing additional tests before making a final decision.

## 4. Discussion

### A Liquid Biopsy for Detecting Early Ovarian Cancer Is Possible

Ovarian cancer is the most lethal of all gynecologic malignancies. Globally, OC is the seventh most diagnosed and the eighth leading cause of cancer-related mortalities among women [1]. To date, no effective cancer screening for early OC exists. Currently, the most frequently used screening tools are transvaginal ultrasound using CA125 as a biomarker. CA125 is a well-known oncomarker in OC and is often elevated in women with advanced stages of OC [61]. It is also associated with non-cancerous diseases, like heart disease, and benign ovarian disorders [62]. In a recent large-scale UKCTOCS study [63] carried out from 2001 to 2014, multimodal screening (MMS) including CA125 and transvaginal ultrasound screening failed to significantly reduce mortality when compared with an unscreened group of patients. Significant differences were noticed only if prevalent cases were excluded [63]. Other studies have demonstrated that screening for ovarian cancer using CA125 and transvaginal ultrasound has low positive predictive values and false positive rates [64]. However, the liquid biopsy test discussed here has the potential to fill this unmet need and become the technology needed for a more effective and sensitive tool for the early diagnosis of ovarian cancer. The liquid biopsy (serum) test using novel G-NBS technology successfully demonstrated the ability to differentiate between groups of healthy human subjects, early-stage OC patients, and metastasizing OC patients when comparing the protease activities measured for each group with the other groups.

G-NBSs are composed of explosion graphene linked to fluorescently labeled consensus sequences (oligopeptides) using tetrakis(4-carboxyphenyl)porphyrin (TCPP) as a fluorescent dye and few-layer explosion graphene as a highly effective quencher. When the targeted protease is not present, a very low fluorescent intensity is measured from the G-NBS due to the string-quenching effect between graphene and TCPP. Once the protease being targeted is present in the serum, the oligopeptide tethered to G is cleaved, which allows the fluorophore to escape from the graphene.

This leads to an increase in the fluorescence signal measured. A panel of G-NBSs with six proteases was tested with a total of 146 serum samples obtained from ovarian cancer patients (localized (n = 46), metastatic (n = 50) and healthy patients (n = 50). Our results demonstrate that all six proteases, MMP3, MMP24, MMP28, CTSK, ADAM10/12, and ADAM15, showed statistically significant differences when comparing fluorescence intensity measurements from the localized OC patient group with both the metastatic OC and the healthy control groups; hence, these results indicate that this panel can be used to screen patients and diagnose ovarian cancer at an early stage. Moreover, five out of the six protease biomarkers (all except ADAM15) had strong statistically significant differences when comparing metastatic OC with healthy control patients. In addition, the advantages of the proposed explainable AL framework for the early detection of OC are multifold. Firstly, AL is employed to improve label efficiency by providing flexibility to train the decision models using a limited number of strategically selected labeled samples, compared with traditional supervised learning approaches. It also incorporates the feature relevance technique to explain the predictions of corresponding decision models. This provides a feature relevance score for all the features corresponding to a sample under consideration. This information aids clinicians in assessing the significance of various proteases during medical diagnosis and perceiving the lack of confidence in model predictions, thereby enabling them to consider the necessity of additional tests before reaching a definitive decision.

It is not uncommon to have machine learning (ML) models identify important predictive features that are typically missed if one focuses only on p-values from classical statistical analysis. That is the case with ADAM17, uncovered to have significant predictive power by the ML model. P-values have been shown to have several limitations, particularly in analyzing complex, high-dimensional biological datasets [65]. One major drawback is that *p*-values rely on strong assumptions about data distributions and independence, which may not hold in real-world scenarios. Additionally, p-values only provide binary significance testing, offering limited predictive power and failing to capture nuanced relationships within data. They are also highly sensitive to sample size. Small sample sizes may lead to unreliable results (high variance), while large sample sizes can make even trivial effects statistically significant [66,67]. Furthermore, p-values do not quantify effect sizes or practical significance, making them inadequate for decision making in complex applications [68,69]. In contrast, modern ML models can handle large, multi-dimensional datasets, learn non-linear relationships, and provide probabilistic predictions with confidence scores, offering a more comprehensive approach to data-driven insights.

In comparison with current liquid biopsy approaches in solid tumors, which are based on (exosome-based) circulating tumor DNA [14], circulating tumor cells [70,71], cell-free RNA [71], and tumor-educated platelets (TEPs) [70], explosion graphene-based nanobiosensors (G-NBSs) are inexpensive (<USD 10 per triplicate measurement) and have superior limits of detection (LOD; sub-femtomolar protease activities; <10^−15^ mol/L). Currently, genetic tests can reveal the potential of developing a disease; however, our G-NBS technology can help predict when the disease will start. Therefore, the combination of both genetic and protease activity G-NBS tests could be used together to monitor high-risk group patients. It should be noted that most women can develop ovarian cancer without being at high risk; however, several factors may increase their risk of developing the disease during their lifetime, including age (>62 years), family history of ovarian cancer, inherited mutation in the DNA damage repair genes *BRCA1* (breast cancer gene 1), and *BRCA2* (breast cancer gene 2), genetic mutations associated with Lynch syndrome, use of hormone replacement therapy, history of endometriosis, and reproductive factors [72,73,74,75,76,77]. Hence, this study is an important step toward the development of less invasive and more sensitive and accurate technologies for OC screening.

It should be noted that this study was concerned with epithelial ovarian cancer only. We anticipate that ovarian germ cell and stromal cell tumors will have different protease signatures.

## 5. Conclusions

This study demonstrates the promising potential for feasible, non-invasive, cost-effective, and efficient screening technology for the early detection of ovarian cancer by means of a liquid biopsy. Currently, genetic tests can reveal the potential of developing a disease, while our G-NBS technology can indicate when the disease actually starts. Therefore, the combination of both genetic and protease activity testing can be used together to monitor high-risk group patients for ovarian cancer.

## Figures and Tables

**Table 1:** Biomarkers identified in NCBI GEO database with corresponding GEO p-values and designed consensus sequences.

GEO *p*-Value	Gene	Oligopeptide Sequence
9.040 × 10^−10^	MMP3	GAG RPFS-MIMG AG
1.130 × 10^−09^	MMP28	GAG MAPK-HKEM AG
2.980 × 10^−09^	CTSK	GAG AKLK-AENN AG
4.090 × 10^−08^	MMP24	GAG NSFG-LRFG AG
3.450 × 10^−07^	ADAM15	GAG AGSH-TTHG AG
4.540 × 10^−06^	ADAM10/12	GAG HSQA-VKSQ AG
1.030 × 10^−08^	ADAM17	GAG LAQA-VRSS AG

**Table 2:** Biomarkers identified in NCBI GEO database with corresponding GEO *p*-values and designed peptide sequence.

Expressed Gene	Localized vs. Control	Metastatic vs. Control	Localized vs. Metastatic
**MMP3**	1.3182 × 10^−15^	8.9394 × 10^−20^	8.2517 × 10^−11^
**MMP24**	7.2331 × 10^−06^	3.9465 × 10^−13^	7.9569 × 10^−05^
**MMP28**	7.8515 × 10^−08^	3.8994 × 10^−13^	0.01021
**CTSK**	3.4749 × 10^−11^	2.9748 × 10^−23^	4.8277 × 10^−09^
**ADAM10/12**	2.0938 × 10^−06^	7.1264 × 10^−22^	1.5586 × 10^−08^
**ADAM15**	1.0166 × 10^−06^	0.20636	0.00753
**ADAM17**	0.13042	0.27206	0.41652

*Significant p*-value < 0.05 highlighted in gray.

**Table 3:** Confusion matrix with hierarchical framework as base decision model.

		Predicted Class		
		Healthy	Localized	Metastatic
True class	Healthy	**47**	1	2
	Localized	3	**42**	1
	Metastatic	0	**2**	**48**

**Table 4:** Confusion matrix with 2-layer neural network as base decision model.

		Predicted Class		
		Healthy	Localized	Metastatic
True class	Healthy	**47**	2	1
	Localized	0	**44**	2
	Metastatic	1	2	**47**

## Data Availability

The primary data will be made available (Excel) from Drs. Covarrubias-Zambrano and Bossmann upon request.
